# Gut Inflammatory Diseases, Infection, and Nutrition

**DOI:** 10.1155/2018/7160859

**Published:** 2018-07-26

**Authors:** Helieh S. Oz, Sung-Ling Yeh, Amedeo Amedei

**Affiliations:** ^1^University of Kentucky Medical Center, Lexington, USA; ^2^Taipei Medical University, Taipei, Taiwan; ^3^University of Florence, Florence, Italy

There is a strong link between inflammation, infectious agents, and nutritional status. Millions of people suffer from chronic inflammatory diseases, and the incidence has significantly increased in recent years. Inflammation is a multifactorial biological and immunological response to different injuries. Inflammation is initiated by several stimuli such as pathogens, chemical irritants, nutritional imbalance, and different cell injures. Inflammation is required in the body process of healing. But, chronic inflammation can cause severe and irreversible complications. Different chronic inflammatory diseases include hepatic, colitis, gastrointestinal, and neurodegenerative. Divers microbial (e.g., *Helicobacter*, *Campylobacter*, *Clostridium*, and *Mycobacterium*), parasitic (e.g., protozoa, helminthes, and flatworms), and viral (HPV, norovirus, and hepatitis B and C) are linked with chronic inflammatory responses. Additionally, nutrition imbalance and specific nutrients may influence immune response, modulating infections, and inflammatory responses.

Crohn's and ulcerative colitis are chronic inflammatory bowel disease (IBD) and progressive complication with a dysregulated gastrointestinal mucosal immune response in patient toward intestinal microbiota. Pediatrics often manifest with more severe Crohn's complication than adults. Crohn's patients may eventually develop strictures and malignancies. IL-17-A-producing T helper cells (Th17) have a key role in provoking progression of disease by production of proinflammatory cytokines which in turn required for regeneration and protection of epithelial cells. Crohn's patients have increased production of IL-17 by T helper cells and higher IL-17 mRNA expression at the mucosal level and increased numbers of Th17 cells are associated with endoscopic definition of disease activity. Unlike previous studies, A. Dige et al. concluded that anti-TNF*α* antibody therapy has no effect on the mucosal levels of IL-17A, IL-21, and IL-22 or LP T cell production during early treatment (first 4 weeks) in Crohn's disease.

Composition of commensal microbiota can influence autoimmune disease progress and persistence. The intestinal microbiota is involved in triggering the immune system and leading to intestinal inflammation. IBD patients suffer from a dysbiosis, with decrease in diversity and abundance of some beneficial commensal bacteria. For instance, significant reductions have been reported in *Bifidobacteria* and *Lactobacillus* in the IBD patients' fecal content suggesting a therapeutic application to normalize the gut flora in autoimmune patients using probiotics. Recent study report that gut microbial can translocate from gut into the organs as gut barrier compromises and pathogenic T helper cells are evident in the gut, liver, and lymphoid organs. Similarly, microbial are also found in hepatic biopsies of autoimmune patients and not in healthy counterparts. Bifico is a probiotic mixture of *Lactobacillus*, *Bifidobacterium*, and *Enterococcus*. Previous studies have demonstrated Bifico to improve colitis and colitis-associated malignancy, pouchitis, diarrhea, and gastritis in models. In addition, clinical trials have revealed therapeutic application for Bifico in Crohn's and ulcerative colitis patients. Bifico has been shown to elevate the expression of colonic TJs and promote intestinal epithelial barrier function in IL-10-deficient mice. In vitro experiments also showed that Bifico, or single probiotic strains *(Bifidobacterium*, *Lactobacillus*, or *Enterococcus)*, increases transepithelial electrical resistance and the expression of TJs in *Escherichia coli*-treated Caco-2 monolayers. Bifico significantly inhibited the secretion of proinflammatory cytokines and reduced bacterial invasion and combination of probiotics were more pronounced than single-strain probiotics. Bifico was shown to have anti-inflammatory effect to expand Tregs in mesenteric lymph nodes and disturbance of Th1/Th2 cytokines in the colonic mucosa of TNBS-induced colitis mice. However, the effect of Bifico on the Tregs in intestinal tissue and peripheral blood has not been reported. These experimental studies were performed in active colitis induced with dextran sulfate sodium (DSS) to explore the therapeutic effects of Bifico. Here, Y. Zhang et al. used DSS-active colitis model to investigate Bifico effect associated with local and systemic immune responses. This study explored the protective effect of Bifico pretreating on subsequent intestinal inflammation.

Acute pancreatitis (AP) is an acute and life-threatening inflammatory disease that commonly damages peripancreatic tissues and other distant organs. A consistent percentage (about the 25%) of patients with severe acute pancreatitis (SAP) develops into infected pancreatic necrosis and persistent organ failure, contributing the most to AP mortality. In fact the SAP, due to excessive release of inflammatory factors and increased oxidative stress response, can cause distant organ damage, especially acute lung injury. In addition, currently, there is no effective therapeutic strategy for AP. For this purpose, Y. Li et al. used Cae-induced mild AP (MAP) model and L-arginine-induced SAP model, to investigate the role of naringenin (Nar) in AP and the accompanying organ dysfunctions in mice as well as the underlying mechanisms. Nar is a type of flavonoid, with anti-inflammatory properties, organ-protective effects, and antioxidative functions. They observed that the serum levels of amylase, lipase, and cytokines and the malondialdehyde (MDA) levels of pancreatic tissue were significantly decreased in both MAP and SAP models after Nar treatment. In contrast, glutathione peroxidase, glutathione reductase, glutathione-S-transferase, total sulfhydryl, and nonprotein sulfhydryl were markedly increased both in MAP and SAP after Nar treatment. In addition, the injury in pancreatic and pulmonary tissues was markedly improved as evidenced by the inhibited expression of myeloperoxidase, nod-like receptor protein 3, and interleukin-1*β* as well as the enhanced expression of nuclear factor erythroid 2-related factor2/heme oxygenase-1 in pancreatic tissues. So, they concluded that Nar exerted protective effects on Cae-induced MAP and L-arginine-induced SAP in mice, suggesting that Nar may be a potential therapeutic intervention for AP.

Gut microbiota (GM) plays several crucial roles in host physiology, influencing different relevant functions. GM diversity is affected by diet and influences metabolic and immune functions of the host's physiology. Consequently, a dysbiosis may be the cause or at least may lead to the progression of various pathologies such as infectious diseases, gastrointestinal cancers, IBD, and even obesity and diabetes. Therefore, GM is an appropriate target for nutritional intervention to improve health and phytochemicals (that can influence GM) have recently been studied as adjuvants for the treatment of obesity and inflammatory diseases. L. Carrera-Quintanar et al. discussed the most recent evidence indicating a relationship between the effects of different phytochemicals and gut microbiota, affecting obesity and/or inflammation. Authors focused on the effect of approximately 40 different phytochemical compounds, candidates for the treatment of obesity and inflammatory diseases. They concluded that several issues need to be resolved before natural products can be effectively translated into the clinic. With regard to the best source of bioactive molecules, the following aspects should be considered: (a) whether it is better to acquire them directly from diet food or from pharmacological sources and (b) whether they should be used alone or as a cotreatment in combination with approved drugs. Therefore, it is urgent to develop specific clinical trials. In addition, disadvantages of commercial nutraceutics' preparations include the high variability in formulations, as well as the dosage quantification and the different means of administration. Finally, critical investigations are required to optimize these phytochemical formulation and dosages for possible future administration.

The digestive system plays an important part in pathogenesis of infection by the human immunodeficiency virus (HIV), which can infect hepatocytes, Kupffer cells, and hepatic stellate cells, inducing the production of inflammatory cytokines and favoring hepatic steatosis. Circulating levels of different hepatic proteins (e.g., albumin, prealbumin, and transferrin) decrease, increasing the mortality risk in AIDS patients. L. Xu et al. evaluated whether the level of butyrylcholinesterase (BchE) could be associated with the progression/prognosis of AIDS patients. Evaluating a cohort of 505 patients, the associations between BChE level and CD4 count, WHO stage, body mass index, and C-reactive protein level, the authors concluded that BChE level is associated with HIV/AIDS severity and is an independent risk factor for increased mortality in AIDS patients.

Surgery is elective treatment for colorectal malignancy, yet the morbidity rate following colorectal resection remains as high as 24%–43%. Some of these postsurgical complications include tissue adhesions at the site of surgery, infections, anastomotic leakage, impaired bowel movement, and malfunction as transient or prolonged postsurgical ileus if not resolved after 5 days or recurrent after recovery. These complications can delay recovery and increase the length of hospitalization and acquired infections and medical expenses. An effective biomarker to predict postsurgical ileus and other complications can be useful for recovery in these patients. G.S.A. Boersema et al. investigated a prospective cohort trail for the association between the inflammatory cytokines and the postoperative complications. The authors studied 47 patients from which 34 (72%) recovered. From 13 patients (28%) who developed postsurgical ileus, 8 (20%) recovered after 5 days and 5 patients (10%) developed recurrent disease. The authors discuss the association of different inflammatory cytokines involvement with postsurgical complications and reason IL-6 changes to predict the infectious complications but not postsurgical ileus after colorectal surgery. They concluded that IL-6 may be promising candidate to assist an early detection of the infections following surgeries.

Maresins, a group of lipid mediators, are biosynthesized from docosahexaenoic acid which displays strong anti-inflammatory and proresolving activity. Resolution of inflammation is an active and highly regulated cellular and biochemical process required to protect against inflammatory complications. S. Tang et al. review the biological actions, pathways, and mechanisms of maresins and their roles in the resolution of inflammation in various disease conditions including lung disease, vascular disease, obesity, diabetes, and IBD. Authors concluded that maresins may prevent neutrophil infiltration, enhance macrophage phagocytosis, inhibit nuclear factor-*κ*B activation, and stimulate tissue regeneration. Similar studies may provide new directions to discover maresin-related stable analogues to control inflammation in the future.

IBD is a multifactorial inflammatory disease of the intestine. Diet has long been suspected to contribute to the IBD development and the Western dietary pattern, which is high fat, high n-6 polyunsaturated fatty acids (PUFA), is associated with an increased IBD risk. N-3 PUFAs contain mostly fish oil and have anti-inflammatory properties. C. Charpentier et al. investigated the dietary influence of fatty acid composition on 2,4,6-trinitrobenzen sulfonic acid- (TNBS-) induced colitis. Rats were fed with diets varying in n-3/n-6/n-9 ratio to reproduce dietary pattern from a pragmatic to a Western diet. There were 4 groups with n-3/n-6/n-9 ratio 1 : 4 : 16, 1 : 1 : 4, 1 : 16 : 16, and 1 : 4 : 24, respectively. n-3/n-6/n-9 ratio 1 : 4 : 16 is recommended as a well-balanced control diet, and 1 : 1 : 4 was a target by dietary advice in a Japanese clinical trial for IBD patients. 1 : 16 : 16 is considered comparable to Western diet, and ratio 1 : 4 : 24 is comparable to the Mediterranean diet. The results showed that compared to the control diet, n-3 polyunsaturated fatty acids-rich diet significantly decreased colon-inducible nitric oxide synthase, cycloxygenase-2 expression, IL-6, and leukotriene B4 production. The authors concluded that n-3 diet group which showed n-3/n-6 ratio equals to 1 attenuated inflammatory markers in the colon that may contribute to partially limit colitis genesis.

Formononetin is an isoflavone compound that has been reported to possess anti-inflammatory properties. D. Wu et al. investigated the effects of formononetin on DSS-induced acute colitis in vivo and in vitro on tumor necrosis factor-*α*-induced human colonic cell injury models. Mice with colitis were intraperitoneal injected with different dosages of formononetin. The main findings showed that formononetin administration relieved clinical symptoms of colitis, mitigated colonic epithelial cell injury, and upregulated the levels of colonic tight junction proteins ZO-1, claudin-1, and occluding. In the in vitro study, the formononetin prevented acute injury of human colonic cells by increasing colonic tight junction proteins and decreasing inflammatory cytokine expression. The mechanism may partly be associated with NLRP3 inflammasome signaling inhibition, as the NLRP3 pathway protein levels including NLRP3, ASC, and interleukin-1*β* were downregulated in a dose-dependent manner, in vivo and in vitro, when formononetin was administered. The authors concluded that formononetin could protect colonic epithelial cells from injury to relieve the disease severity of colitis and may have potential to be used in the clinical prevention and treatment of IBD in future.

Angiostrongylosis is an important food-borne diseases and eosinophilic encephalitis in humans, caused by rat gut nematode (rat lungworm). Outbreaks of eosinophilic meningitis have been reported due to the consumption of infected raw snails and vegetables' juice. Angiostrongylus invades the central nervous system and causes neurons' demyelination, eosinophilic encephalitis, and meningoencephalitis. An inflammatory response and surge of cytokines such as IL-17 have been detected in the central nervous system. IL-17 can induce Act1-mediated signaling cascades in CNS resident cells (astrocytes, oligodendrocytes, and neurons) might coordinately mediate CNS inflammation, demyelination, and neurodegeneration. But the mechanisms by which IL-17 involves in the demyelination caused by this nematode is not investigated. F. Ying et al. explore the role of IL-17A on the demyelination and introduce IL-17A-neutralizing antibody to protect against demyelination caused by the parasite as a possible therapeutic option in angiostrongylosis. In addition, iNOS inhibition is the possible mechanism for the therapeutic effect. This study provides a new potential therapeutic alternative for demyelination caused by *Angiostrongylus cantonensis*.

Alcohol consumption in excess causes extensive liver injuries of fatty liver which progresses to hepatitis, fibrosis, cirrhosis, and hepatocarcinoma. Ethanol increases NADH/NAD^+^ ratio and promotes fatty acid synthesis and lipid accumulation in liver cells. Further, it causes excessive oxidative stress and increases CYP2E1 activity. In addition, ethanol increases endotoxin bypass from leaky gut which triggers Kupffer's cell activation and inflammatory processes. There is an emerging theory that chronic ethanol abuse damages the tight junction structure in intestinal epithelial cells results in bacterial translocation from the intestines into the in vivo circulation to induce hepatic inflammation. Indeed, patients with alcoholic liver disease have higher levels of endotoxin and intestinal barrier disturbances caused by ethanol are the major source of endotoxemia in these patients. Different source of dietary fat can effect progression of liver injury as diets rich in saturated fatty acids or medium chain triglycerides protect against ethanol-induced liver injury in rodents. In contrast, polyunsaturated fatty acids can provoke liver injury. However, there were some limitations of these previous studies such as only one type of fat was used in each experimental diet, but the effects on other organs or tissues were not explored. Studies have demonstrated that fish oil, which is rich in n-3 polyunsaturated fatty acids, eicosapentaenoic acid, and docosahexaenoic acid, can provide immune regulation, vascular protection, and lipid metabolism modulation. However, the mechanism by which oil can modify intestinal integrity in alcoholic liver disease is not well understood. Further, some studies reveal fish oil and olive oil to improve the fecal microbiota under ethanol exposure, but the effects on intestinal pathological changes in ethanol-fed rats are still unclear. Y.-W. Chien et al. explored whether fish oil can provoke hepatoprotective effects in ethanol-fed rats by means of maintaining the epithelial barrier function in the intestines and further inhibiting endotoxin in circulation. Authors concluded that the chronic ethanol can elevate plasma endotoxin concentrations and trigger inflammatory responses which can result in liver injury. Substitution of fish oil for olive oil inhibited the appearance of endotoxin in the circulation under ethanol exposure; thus, it decreased inflammatory responses and exerted a hepatoprotective potential in rats under chronic ethanol feeding. However, the mechanism of decreased plasma endotoxin levels by fish oil supplementation alone might not be enough to improve intestinal structural integrity.

About 30–40% of chronic inflammatory disease patients use some form of complementary and alternative medicine, including Chinese traditional herbal therapies. It is estimated that 12 million tons of herbal wastes is produced each year by about 1,500 Chinese traditional herbal medicine enterprises in China. During process, the active ingredients are extracted from plants, and the waste which still contains about 30%–50% of the medicinal active ingredient is buried or burned which becomes major source of environmental pollutant in water and in air. Fermentation by digestive enzymatic reaction utilizing cellulase, protease, pectinase, and lipase can degrade plant cell wall and expose intercellular organelles to assist in extraction of active ingredients. In addition, certain probiotics can improve digestive process and protect against malabsorption, malnutrition, and diarrhea. Antibiotic-associated diarrhea is a frequent side effect caused by altered gut microbiota which supports pathogen growth. F. Meng et al. used probiotics to ferment the herbal residues in Jianweixiaoshi, a mixture of herbal remedy used for diarrhea, and the reason for this compound may become as therapeutic potential against antibiotic-associated diarrhea, as well as to reduce the waste products from herbal residues produced by traditional herbal medicine enterprises.

Flowers of *Osmanthus fragrans (O. fragrans)* and *Chrysanthemum morifolium (C. morifolium)* are commonly used as folk medicine and additives for tea and beverages. The active components isolated from these flowers contain many phenolic compounds that have been shown to have anti-inflammatory and antioxidant properties. Lipotoxicity occurs when excessive harmful lipid accumulates in cells leading to cellular dysfunction and disruption of tissue function. Lipotoxicity plays a critical role in the pathogenesis of nonalcoholic fatty liver disease and renal diseases. P.-J. Tsai et al. investigated the effects of methanolic flower extracts of *O. fragrans* and *C. morifolium* against free fatty acid-induced lipotoxicity in hepatocytes and renal glomerular mesangial cells. The results showed that both extracts inhibited free fatty acid-induced hepatocyte triglyceride accumulation and suppressed mRNA expression of inflammatory cytokines when hepatocytes were stimulated with lipopolysaccharide-treated monocytes. Also, flowers extracts of *O. fragrans* and *C. morifolium* effectively inhibited oleate-induced cellular lipid accumulation and overexpression of fibronectin in mesangial cells. The authors concluded that these flower extracts possess hepato- and renal-protective activity by inhibiting hepatic fat load and inflammation and mesangial extracellular matrix formation. These findings imply that flowers of *O. fragrans* and *C. morifolium* may have potential to protect against nonalcoholic steatohepatitis and renal fibrosis.

*Helieh S. Oz* *Sung-Ling Yeh* *Amedeo Amedei*

